# P45 A qualitative study exploring the experiences, views and scope of practice of pharmacists working in infectious disease services in Irish hospitals

**DOI:** 10.1093/jacamr/dlaf118.052

**Published:** 2025-07-14

**Authors:** T M Barbosa, L Healy, P Maher, L Quigley, A Fleming

**Affiliations:** Pharmaceutical Care Research Group, School of Pharmacy, University College Cork, Cork, Ireland; Pharmaceutical Care Research Group, School of Pharmacy, University College Cork, Cork, Ireland; Pharmaceutical Care Research Group, School of Pharmacy, University College Cork, Cork, Ireland; National Clinical Programme for Infectious Diseases, Health Service Executive, Dublin, Ireland; Pharmaceutical Care Research Group, School of Pharmacy, University College Cork, Cork, Ireland; Pharmacy Department, Mercy University Hospital, Cork, Ireland

## Abstract

**Background:**

Infectious diseases (ID) pose ongoing challenges within healthcare, requiring a multidisciplinary approach to patient management. ID pharmacists play an essential role in the provision of a variety of ID services, ensuring the safe and appropriate delivery of pharmaceutical care. While insights into this role have been described internationally, the role of the ID pharmacist remains underdefined in the Irish context.

**Objectives:**

To explore the experiences, roles and scope of practice of pharmacists providing ID services in Irish hospitals.

**Methods:**

Semi-structured interviews were conducted with pharmacists working in ID services in Irish hospitals in November 2024. Thematic analysis was used to identify themes in interview transcripts. Ethical approval and written informed consent were obtained.

**Results:**

Sixteen pharmacists were recruited from twelve different hospitals across Ireland. Within the sample there was variety in gender, age, years of experience, and roles. Predominant themes included the broad scope of the role and responsibilities of ID pharmacists, the positive impact of ID pharmacists, barriers and challenges in the role such as staffing shortages, lack of facilities and workload, future aspirations and comparison to international standards, including the role of the pharmacy technician. Pharmacists play a crucial role in medication management, patient counselling, and multidisciplinary collaboration. However, inadequate formal recognition, the absence of structured ID training, the importance of interprofessional collaboration and management of ID patients in hospitals without a specific ID service were raised. Pharmacists expressed a strong desire for independent prescribing rights and pharmacist-led ID clinics, aligned with international models.

**Conclusions:**

This study provides an in-depth insight into the scope and impact of the pharmacist in ID services in Ireland. While pharmacists contribute substantially to ID patient care, challenges around staffing levels, resource limitations and workload pressures that limit their full integration into healthcare teams need to be addressed. Expanding their role through policy changes, structured training, and independent prescribing could enhance patient outcomes and align Ireland with international best practices. These findings should inform future service development to advance the pharmacist role in ID services and ensure integration of the role in the health service.

